# Nutritional Intervention Restores Muscle but Not Kidney Phenotypes in Adult Calcineurin Aα Null Mice

**DOI:** 10.1371/journal.pone.0062503

**Published:** 2013-04-30

**Authors:** Kirsten Madsen, Ramesh N. Reddy, S. Russ Price, Clintoria R. Williams, Jennifer L. Gooch

**Affiliations:** 1 Atlanta Veterans Administration Medical Center, Decatur, Georgia, United States of America; 2 Department of Medicine/Division of Nephrology, Emory University School of Medicine, Atlanta, Georgia, United States of America; 3 Department of Cardiovascular and Renal Research, University of Southern Denmark, Odense, Denmark; UCL Institute of Child Health, United Kingdom

## Abstract

Mice lacking the α isoform of the catalytic subunit of calcineurin (CnAα) were first reported in 1996 and have been an important model to understand the role of calcineurin in the brain, immune system, bones, muscle, and kidney. Research using the mice has been limited, however, by failure to thrive and early lethality of most null pups. Work in our laboratory led to the rescue of CnAα−/− mice by supplemental feeding to compensate for a defect in salivary enzyme secretion. The data revealed that, without intervention, knockout mice suffer from severe caloric restriction. Since nutritional deprivation is known to significantly alter development, it is imperative that previous conclusions based on CnAα−/− mice are revisited to determine which aspects of the phenotype were attributable to caloric restriction versus a direct role for CnAα. In this study, we find that defects in renal development and function persist in adult CnAα−/− mice including a significant decrease in glomerular filtration rate and an increase in blood urea nitrogen levels. These data indicate that impaired renal development we previously reported was not due to caloric restriction but rather a specific role for CnAα in renal development and function. In contrast, we find that rather than being hypoglycemic, rescued mice are mildly hyperglycemic and insulin resistant. Examination of muscle fiber types shows that previously reported reductions in type I muscle fibers are no longer evident in rescued null mice. Rather, loss of CnAα likely alters insulin response due to a reduction in insulin receptor substrate-2 (IRS2) expression and signaling in muscle. This study illustrates the importance of re-examining the phenotypes of CnAα−/− mice and the advances that are now possible with the use of adult, rescued knockout animals.

## Introduction

Calcineurin is a multi-subunit phosphatase that requires the association of a regulatory subunit (B), a catalytic subunit (A), and the binding protein calmodulin to be functionally active [1]. There are three isoforms of the catalytic subunit - α, β, and γ - that are the products of three separate genes. The amino acid sequences of all three isoforms are remarkably similar, particularly within the phosphatase domain [1], [2]. However, mice lacking the α or β isoform have very different phenotypes 3–7, suggesting that the isoforms have some independent functions. CnAα−/− mice were first reported in 1996 by Jon Seidman’s laboratory. The principle findings were changes in the brain consistent with hyperphosphorylation of the cytoskeletal protein substrate tau and memory impairment [6]. Interestingly, the mice were still susceptible to immune suppression by cyclosporine and T cell development was only mildly affected by loss of the α isoform [6]. In 2002, Jeffrey Molkentin’s group published their findings of CnAβ−/− mice. In contrast to CnAα−/− mice, which, in addition to hyperphosphorylation of tau, suffer from failure-to-thrive, infertility, and a shortened lifespan, CnAβ−/− mice develop normally and reproduce. However, inspection of their immune system revealed what the group termed “an immune-suppressed-like” phenotype [4]. Specifically, the mice have impaired T cell maturation characterized by a significant deficit in both CD4+ and CD8+ cells. Moreover, isolated T cells exhibit decreased T cell receptor signaling in response to in vitro stimuli that cannot be further reduced by cyclosporine. These data highlight the importance of understanding the differences between CnAα and CnAβ action.

In addition to the immune system, CnAα−/− and CnAβ−/− mice differ in their growth and development. While CnAβ−/− mice have a normal body weight and are fertile, failure to thrive and early lethality of CnAα^−/−^ mice has been noted by several groups including our own [3], [6]–[8]. Recently, we reported that loss of CnAα alters salivary gland function [9]. This finding was particularly significant because it suggested a possible way to intervene in the failure to thrive which was a predominant feature of null pups. Subsequently, we reported that CnAα−/− mice could be rescued by feeding them a modified diet. Null mice quickly regained weight comparable to littermates and maintained a normal body mass into adulthood [9]. These results make it clear that previous reports of CnAα functions were made in the setting malnutrition. The goal of the current study is to re-examine adult, rescued CnAα−/− mice in order to determine what aspects of the phenotype previously reported by our group was the result of CnAα loss and what was due to early nutritional deprivation.

## Materials and Methods

### Animal Models

Calcineurin Aα knockout mice **(**CnAα−/−) mice were created by J. Seidman (Harvard Medical School, Boston, MA) as previously described [6] and have been maintained in our laboratory at the Atlanta Veterans Administration Medical Center. All procedures were carried out in strict accordance with approved IACUC protocol # VA-017. CnAα−/− mice were generated and have been maintained on a mixed genetic background [6]. According to Zhang et al [6], the knockout cloning vector was introduced into 129 embryonic stem cells that were then transferred into C57BL/6J blastocysts and then injected into Black Swiss pseudo pregnant females. Male offspring were mated to Black Swiss or C57BL/6J females. Since 2003, a colony of CnAα mice have been maintained in the Gooch lab using standard brother sister mating protocols. All experiments were carried out with 6–8 week old littermates. Dietary supplementation consisted of previously reported methods [9] with some modifications. Briefly, 0.16 g of PancreVed Powder (Vedco, Encinitas, CA) was dissolved in 30 ml peanut oil and then 500 ul was applied to approximately 500 grams of moistened, standard rodent chow. CnAα−/− mice and littermate controls were supplied with food cups beginning at approximately 14 days of age and continued throughout the life of the animals. All data were obtained using control and knockout mice fed the same supplemental diet *ad libitum*.

### Kidney Function

Kidney function was assessed by measurement of serum blood urea nitrogen (BUN) levels using a Reflotron benchtop chemistry analyzer and approximating glomerular filtration rate (GFR) as previously described [10]. Briefly, serum BUN was divided by urine BUN times urine production rate to approximate nitrogen clearance. Urine was collected over 24 hours in rodent metabolic cages and urine osmolality was determined using a Westco osmometer. In addition, albumin and creatinine were measured in spot urine samples by ELISA assay and the albumin to creatinine ratios were calculated.

### Quantitative Histology

Kidneys were fixed by systemic perfusion with formalin through the left ventricle of the heart at a constant pressure of 100 cm/H_2_O. Perfused kidneys were embedded in paraffin, sectioned at 4 µm and then stained with H&E for routine imaging. Some sections were also stained with Silver stain to visualize matrix proteins. For quantitation of cross sectional area of kidney zones, H&E stained coronal sections through all regions including the tip of the papilla were photographed under light microscopy at x4 magnification. Using ImagePro software (Media Cybernetics, MD), cortex, outer stripe of outer medulla (OSOM), inner stripe of outer medulla (ISOM) and inner medulla (IM) were framed and cross sectional area automatically calculated. Longitudinal axis was defined as a line through the tip of the papilla and perpendicular to the cortical surface. Length of axis was measured using ImagePro software as previously mentioned. For quantitation of glomerular size, H&E stained kidney sections were photographed under light microscopy at identical magnification. The harmonic mean glomerular area was determined using ImagePro software. Area sizes of at least 50 glomeruli per mouse were measured.

### Muscle Histology and Fiber Type Staining

Hindlimb muscles were snap frozen, sliced at 5µM, and mounted on glass slides. Tissue was then incubated for 15 minutes at room temperature with NADH-tetrazolium solution (0.2 M Tris, 1.5 mM nicotinamide adenine dinucleotide (NADH), and 1.5 mM nitro tetrazolium). Coverslides were mounted with Vectashield and fiber types analyzed by light microscopy. 4–6 different sections of muscle were analyzed and a total of 100–115 fibers were characterized per animal. Unstained fibers were designated type IIb, moderately stained fibers IIa/x, and intensely stained fibers type I.

### Western Blotting

Approximately 100 mg of tissue was lysed in TNESV lysis buffer (50 mM Tris-HCl pH 7.4, 2 mM EDTA, 1% NP-40, 100 mM NaCl, 100 mM Na orthovanadate, 100 µg/ml leupeptin, 20 µg/ml aprotonin, and 10^−7^ M phenylmethylsulfonyl (PMSF)). Alternatively, primary muscle cells were plated in 60 mm dishes and allowed to grow to 80–90% confluence and the medium was changed to serum-free medium (SFM) for 24 hours and the cells were treated with insulin (1 ng/ml) for 10 minutes as indicated. Cells were harvested with trypsin-EDTA, pelleted, washed with 1X PBS, and lysed using TNESV lysis. 25 µg of protein was separated by 7.5% SDS-PAGE and proteins transferred to nitrocellulose. The membrane was incubated in 5% milk-TBST (20 mM Tris-HCl, pH 7.6, 137 mM NaCl, 0.1% Tween 20) and then immunoblotted with appropriate dilutions of primary antibodies as specified by the manufacturer. Total and phospho Akt, ERK1/2, GSK-3β primary antibodies were purchased from Cell Signaling Technology (Danvers, MA) and IRS1 and IRS2 antibodies were from SantaCruz Biotechnology (SantaCruz, CA). Membranes were incubated with HRP-conjugated secondary antibody, and proteins were visualized by enhanced chemiluminescence (Pierce, Rockford, IL).

### Statistics

All statistical analyses were completed using GraphPad Prism software (La Jolla, CA). Statistical significance was considered when p<0.05. Unless otherwise stated, all comparisons were two-way ANOVA.

### Ethics

All experiments in this study were approved by the Atlanta Veterans Administration Medical Center Institutional Animal Care and Use Committee protocol #VA-017.

## Results

### Rescue and General Phenotype of CnAα−/− Mice

**Figure 1:**
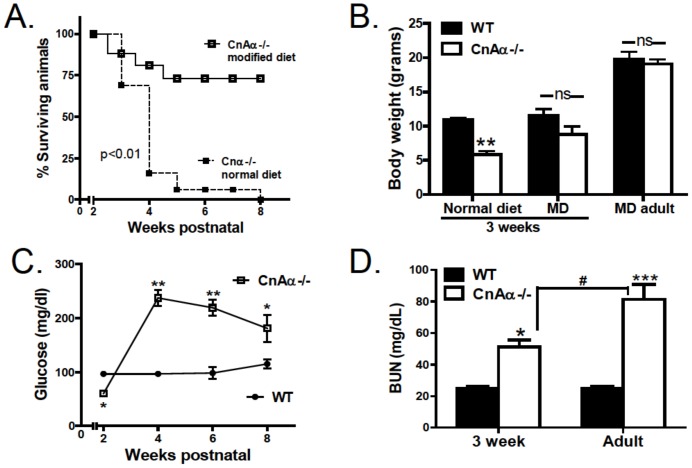
Kidney function of rescued CnAα−/− mice. **A)** Survival curve of CnAα−/− fed a conventional diet (dotted line) and the enzyme supplemented, modified diet (MD) (solid line). Data are derived from breeding records of N = 40–50 mice for each group. **B)** Body weight of WT and CnAα−/− mice at 3 weeks of age fed the normal or MD diets were compared. Likewise, body weights of adult WT and CnAα−/− mice (approx8 weeks of age) were compared. Data shown are the mean body weights of 8 WT and 10 null mice and include both males and females. **p<0.01 compared to WT. **C)** Blood glucose levels were monitored before and up to 8 weeks after supplemental feeding with the MD. Data shown are the mean blood glucose level at each time point of 8–10 mice and include both males and females. *p<0.05 and **p<0.01 compared to WT. **D)** Blood urea nitrogen measurement of 4 wildtype and 4 CnAα^−/−^ mice at 3 weeks and 10 CnAα^−/−^ mice at 8 weeks of age. *p<0.05 and ***p<0.001 compared to WT; # p<0.05.

Mice in this study were rescued by supplemental feeding as previously described [9] with minor modifications (see Methods). CnAα−/− mice and wildtype (WT) littermates were provided a modified diet (MD) beginning at approximately 2 weeks of age; supplemental feeding with the MD was offered to all mice throughout the experiment. 75% of mice fed the MD survived until 8 weeks of age compared to 0% of the mice fed standard rodent chow (**Figure 1A**). **Figure 1B** shows that by three weeks of age, mice fed a normal rodent diet had significantly smaller body mass than WT littermates. However, mice receiving the MD were no longer significantly smaller than WT littermates at 3 weeks and by six weeks of age, body weights of both groups were comparable. Null mice maintained a normal body weight through 4 months of age (data not shown) and both males and females are fertile.

In addition to survival and body weight, blood glucose levels were monitored to assess caloric intake. Previously, we reported that null mice were hypoglycemic [3]. Within 2 weeks of supplementation with the MD, null mice had elevated blood glucose levels which moderated slightly over time but remained significantly elevated compared to WT littermates (**Figure 1C**). Last, **Figure 1D** shows that blood urea nitrogen (BUN) levels continue to increase from 3 to 6 weeks in null mice compared to WT littermates.

Previously, we reported that kidney, heart and liver weights were significantly reduced in null mice [3]. Therefore, we next examined organ weights in rescued adult mice. **Table 1** shows that while liver and heart mass are normalized in rescued null mice, kidney mass remains reduced. Interestingly, spleen mass is increased in rescued null mice compared to WT littermates.

**Table 1:** Organ weights in WT and rescued CnAα−/− mice.

	*Wildtype*	*CnAα−/−*
Body weight (g)	22.9±0.8	21.3±0.7†
Total heart (mg)	106±7	93±6†
Heart/bw	0.48±.02	0.52±.03†
Total kidney (mg)	334±8	**254±10** ** †
Kidney/bw	1.47±.02	**1.20±.03** ** †
Total liver (mg)	883±33	890±41†
Liver/bw	4.13±.12	4.17±.10†
Total spleen	85.0±2	**117±7** **
Spleen/bw	0.37±0.05	**0.56±0.04** **

Data shown are the mean ± SEM of 8 WT and 10 CnAα−/− mice (6–8 weeks of age).

** *p*<0.005 compared to Wt;

† significant change compared to previously published data [3].

### Kidney Development and Function of Rescued CnAα−/− Mice is Abnormal

Renal function was assessed by measuring urea nitrogen in the blood and urine and approximating glomerular filtration rate (GFR) as previously described [10]. Consistent with impaired renal function, GFR is significantly decreased in 8 week old, adult null mice (**Table 2**). 24 hour urine analyses showed that urine output rate and total protein were not changed in null mice. Albumin to creatinine ratios were also not significantly different. As Figure 1 shows, hyperglycemia is a new phenotype of CnAα−/− mice. To further examine this, we examined serum glucose levels in fasting and *ad libitum* fed rescued null mice and in WT littermates. CnAα^−/−^ mice have significantly elevated fasting and fed glucose levels. Mean insulin levels in null mice trend higher but do not reach significance (**Table 2**).

**Table 2:** Kidney function in CnAα−/− and WT littermates.

	*Wildtype*	*CnAα−/−*
BUN (mg/dl)	28.2±1.9	**81.5±9.3** ** †
GFR (mg/dl/min)	6.8±1.0	**1.50±.3** **
Urine output (µl/min)	0.22±.04	0.22±.06
Urine total protein (mg/ml)	19.4±2.0	18.4±3.0
Albumin : creatinine ratio (ACR)	0.43±0.14	0.29±0.10
Fasting Serum glucose (ng/dl)	95.2±3.9	**143.8±13.5** * †
Fed serum glucose (ng/dl)	114.6±4.1	**219.3±22.6** **
Serum insulin (ng/dl)	0.51±.03	0.79±.24

Data shown are the mean ± SEM of 8 WT and 10 CnAα−/− mice (6–8 weeks of age).

* *p*<0.05,

** *p*<0.005 compared to WT,

† compared to previously published data [3].

**Figure 2:**
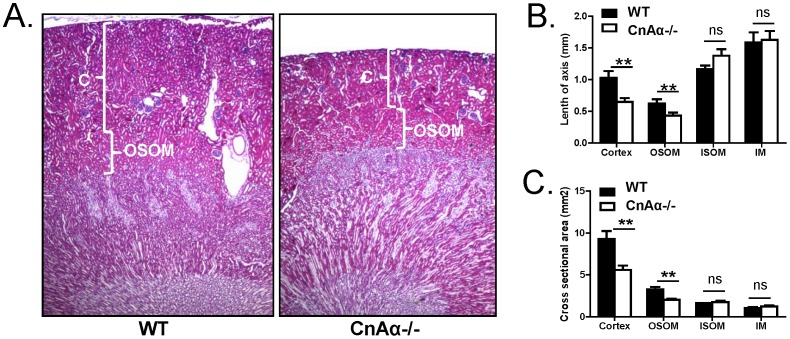
Measurement of kidney regions in CnAα−/− and WT kidneys. **A)** Representative H&E staining of perfusion fixed kidney sections from WT and CnAα−/− mice. Original magnification was 10x. **B)** Quantitation of axonal length measured as a perpendicular line from the tip of the papilla to the cortical surface. Results from WT (N = 5) and CnAα−/− (N = 6) mice are shown. **p<0.01 compared to WT. **C)** Quantitation of cross sectional area of cortex, OSOM, ISOM and IM in WT (N = 5) and CnAα−/− (N = 6) mice. **p<0.01 compared to WT.

Data thus far indicate that despite supplemental feeding with the modified diet, CnAα−/− mice still have impaired renal function and reduced kidney mass. To further characterize the kidney phenotype of rescued mice, we obtained perfusion fixed kidneys and measured the axonal length and cross sectional area of the cortex, outer stripe of the outer medullae (OSOM), inner stripe of the inner medullae (ISOM) and the inner medullae (IM). **Figure 2A** shows a representative image of a kidney section from a WT mouse and a rescued CnAα−/− mouse demonstrating a substantial difference in cortical mass. Quantitation of axonal length and cross sectional area confirms that reduced kidney mass in null mice is due primarily to attenuation of the cortex and OSOM. In contrast, neither parameter is altered in the ISOM or IM (**Figure 2B and 2C**).

**Figure 3:**
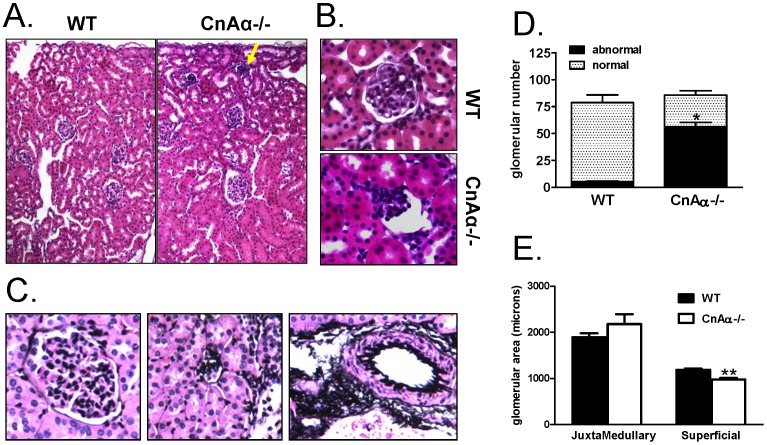
Effect of CnAα loss on glomeruli. **A)** H&E stained WT and CnAα−/− cortical kidney sections. Original magnification was 20x. Arrow indicates abnormal superficial glomeruli. **B)** Superficial cortical glomeruli from WT and CnAα−/− mice; original magnification was 60x. **C)** Silver stained WT and CnAα−/− cortical kidney sections were examined by light microscopy. Original magnification was 20x. **D)** The total number of glomeruli from 5 WT and 6 null mice were counted and scored as normal in appearance or abnormal. *p<0.05 compared to WT. **E)** Glomerular area was determined for both juxtamedullary and superficial glomeruli from 5 WT and 6 null mice. **p<0.01 compared to WT.

Previously, changes in superficial cortical glomeruli were key features of the null phenotype [3]. However, postnatal nutritional deprivation has also been demonstrated to alter glomerular development [11], [12]. Therefore, we examined glomeruli in perfusion fixed kidneys from rescued null mice and WT littermates. **Figure 3A** shows that rescued CnAα−/− kidneys continue to demonstrate a specific defect in late developing, superficial glomeruli (arrow). While juxtamedullary glomeruli are generally normal in appearance, superficial glomeruli are markedly abnormal (**Figure 3B**). Silver staining reveals that glomerular fibrosis is minimal in CnAα−/− sections but markedly increased in the perivascular region (**Figure 3C**) as previously reported [3], [8]. Although the number of glomeruli is not different, the percentage of glomeruli scored as abnormal by two blinded reviewers is significantly increased in CnAα−/− kidney sections (**Figure 3D**). Finally, the mean area of juxtamedullar glomeruli was increased, but the change did not reach significance. However, the area of superficial glomeruli was significantly decreased in null kidneys compared to WT (**Figure 3E**).

### Rescued CnAα−/− Mice have Impaired Insulin Signaling

**Figure 4:**
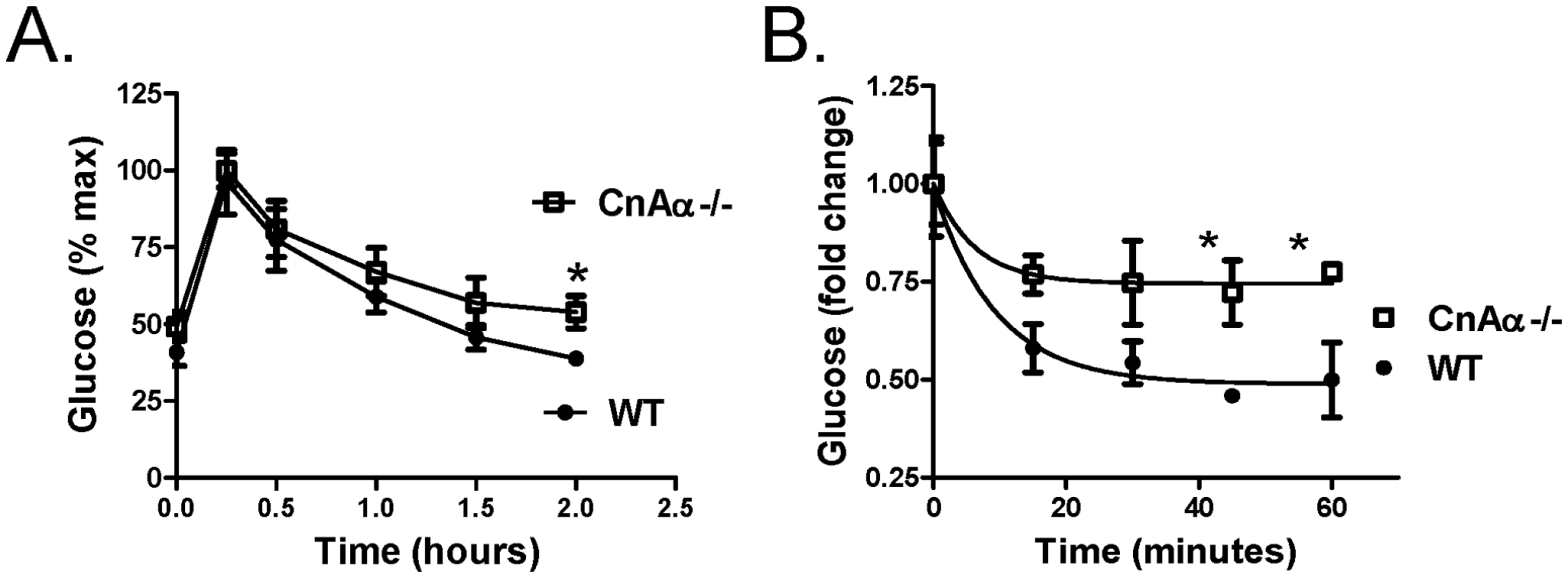
CnAα−/− mice have mild insulin resistance. **A)** Glucose tolerance tests were performed by injecting 1 mg/kg body weight of glucose ip and then monitoring blood glucose levels over the next 2 hours. Data shown are the mean ± SEM of 8 mice per group. *p<0.05 compared to WT. **B)** Insulin sensitivity tests were performed by injecting 1 U insulin ip and then monitoring blood glucose levels over the next 2 hours. Data shown are the mean ± SEM of 8 mice per group. *p<0.05 compared to WT.

The data thus far suggested that early caloric deprivation did not significantly alter the renal phenotype of CnAα−/− mice. However, rescue of the mice did reveal a potential new role for CnAα in regulation of glucose levels. Interestingly, mice that overexpress constitutively active CnAα in the muscle have enhanced insulin responsiveness [13]. Loss of CnAα could, therefore, result in decreased insulin sensitivity. To examine this further, we performed glucose tolerance and insulin sensitivity testing on adult CnAα−/− and WT littermate mice. **Figure 4A** shows that CnAα−/− mice are slightly slower to return to baseline following a glucose challenge. However, the mice had a markedly attenuated response to a direct insulin challenge (**Figure 4B**). Coupled with normal pancreatic histology (data not shown) and slightly elevated circulating insulin levels, the data are consistent with decreased insulin sensitivity in the absence of CnAα.

**Figure 5:**
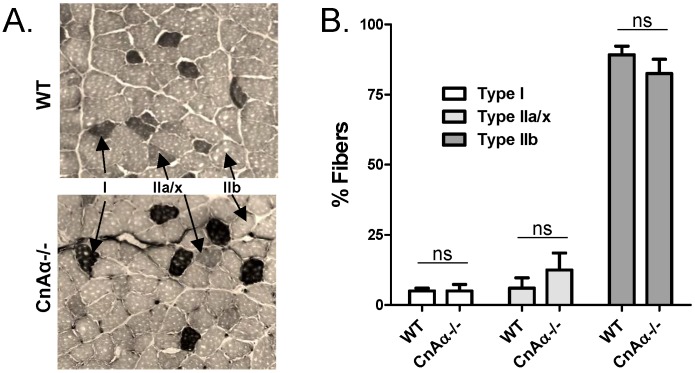
Fiber type distribution of CnAα−/− skeletal muscle is normal. **A)** Gastrocnemius muscle samples were fixed and stained with NADH-tetrazolium. Typically appearing type I, IIa/x, and IIb fibers are indicated with arrows on each panel. Data shown are representative of 6 mice per group. **B)** The distribution of type I, IIa/x, and IIb fibers were quantified and graphed. The data shown are the mean ± SEM of 6 mice per group.

One mechanism for altered insulin sensitivity could be changes in the relative proportions of fast versus slow twitch skeletal muscle fibers which have different levels of glucose utilization. Previously, CnAα−/− mice were reported to have significantly fewer Type I and Type IIa/x muscle fibers compared to WT littermates [14]. Therefore, we re-examined the distribution of Type I, IIa/x, and IIb muscle fibers in hindlimb skeletal muscle from WT and CnAα−/− mice. **Figure 5A** shows a representative result from NADH-tetrazolium staining of fixed gastrocnemius muscle. Quantitation of the fiber types reveals that there are no significant differences in fiber type distribution in the gastrocnemius muscle of rescued null mice compared to WT controls (**Figure 5B**).

**Figure 6:**
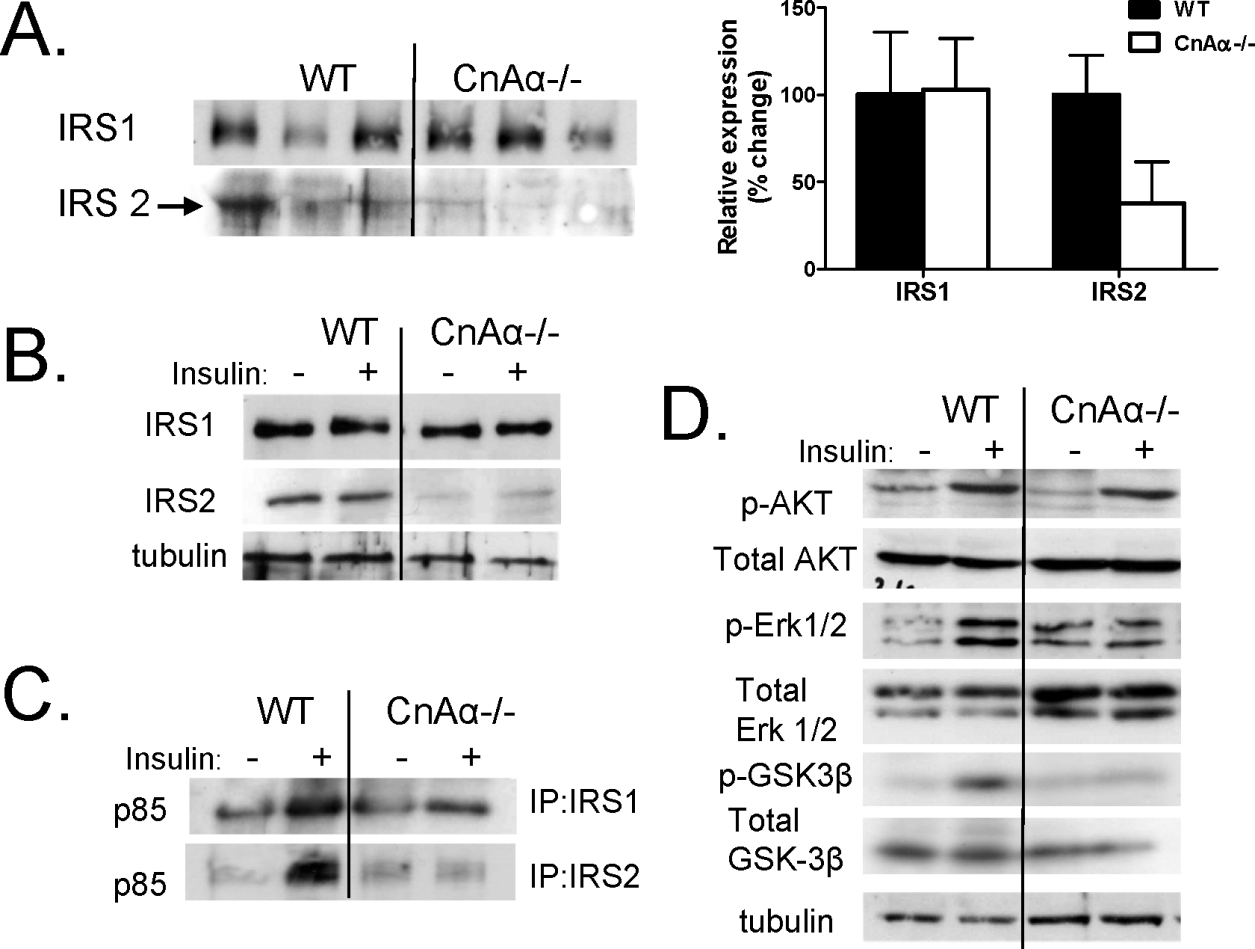
IRS2 expression and signaling is decreased in CnAα−/− muscle cells. **A)** Expression of IRS1 and IRS2 was determined in WT and CnAα−/− muscle lysates by western blotting. Each lane is tissue from a different animal. Data were semi-quantitated and graphed. **B)** IRS1 and IRS2 expression in cultured primary WT and CnAα−/− muscle cells with and without insulin treatment was determined by western blotting. Data shown are representative of at least 3 independent experiments. **C)** The association of IRS1 and IRS2 with the p85 catalytic subunit of PI-3kinase in response to insulin was determined by immunoprecipitation with anti-p85 antibody and then western blotting with anti-IRS1 or anti-IRS2 antibodies. Data shown are representative of at least 3 independent experiments. **D)** Activation of downstream signaling targets in response to insulin were compared in WT and CnAα−/− muscle cells by western blotting with total and phospho-specific antibodies as indicated. Data shown are representative of at least 3 independent experiments.

As an alternative to deranged fiber type distribution, we examined expression of key insulin signaling proteins including the insulin receptor substrates 1 and 2 (IRS1 and IRS2) in hindlimb skeletal muscle tissue. **Figure 6A** shows that expression of IRS1 is unchanged in CnAα−/− muscles compared to WT littermates. However, IRS2 expression is substantially decreased. Chronic kidney failure has been described to alter insulin signaling in the muscle consistent with dysfunctional glucose utilization [15], [16]. To determine if changes in IRS2 were the result of renal failure or were a direct consequence of CnAα loss, primary muscle cell cultures were generated from WT and CnAα−/− mice. Cells were treated with insulin for 10 minutes and then the expression of IRS1 and IRS2 as well as downstream signaling events was examined by western blotting. First, similar to muscle tissue, primary muscle cells show a reduction in IRS2 but not IRS1 with loss of CnAα (**Figure 6A**). This suggests a direct role for CnAα in IRS2 expression. Next, activation of IRS1 and IRS2 in response to insulin was investigated by examining association of the p85 catalytic subunit of PI-3Kinase with IRS1 and IRS2 by co-immunoprecipitation. The amount of p85 bound to IRS1 and IRS2 increases in response to insulin in WT cells. However, while p85 association with IRS1 increases in CnAα−/− cells, there is no increase in the amount of p85 with IRS2 in response to insulin (**Figure 6C**). Finally, downstream signaling events were investigated. Phosphorylation of Akt, which is downstream of IRS1, is normal in CnAα−/− cells but phosphorylation of Erk1/2 and GSK-3β are reduced, consistent with defective downstream IRS2 signaling (**Figure 6D**).

## Discussion

Calcineurin Aα null mice were first reported in 1996. Since then, the mice have been used in studies of the brain [7], [17], immune system [6], skin [18], [19], muscle [14], and kidney [3], [8], [20]–[22] (summarized in **Table 3**). In each of these previous studies, null mice were noted to be smaller although the degree reported by different groups varied. Failure to thrive and early lethality limited investigations with the model and may have influenced many of the results. With the rescue of CnAα−/− mice reported earlier this year, we have the opportunity to re-visit experimental areas of interest in adult animals that are not nutritionally deprived. Our first goal was to examine key aspects of kidney development and function previously reported by our group.

**Table 3:** Summary of previously published studies based on CnAα−/− mice.

*System*	*Major findings*	*Reference*
**Bone**	Osteoporosis through altered osteoblast differentiation	[27], [28]
**Brain**	Increased phosphorylation of tau; altered hippocampal depotentiation	[7], [29]
**Immune system**	Slight decrease in IL-2 levels, minor or no change in T and B cell development and function	[6]
**Kidney**	Altered cortical development, increased fibrosis, decreased renal function	[8], [20]
**Muscle**	Decreased type I and IIa/x muscle fibers	[14]
**Skin**	Increased cell death in supra-basal epithelial layers	[30]

The data in this study clearly demonstrate that, contrary to what we and others previously concluded, loss of CnAα does *not* cause pervasive defects in maturation and development. Rather, the most significant effect on development is impaired vesicle secretion in the salivary gland that leads to decreased enzyme levels during the peri-weaning period and, subsequently, starvation. Supplementation with the modified diet aids early digestion and restores normal weight gain and body mass. In rescued adult mice, we find that most organ deficits previously noted are normalized. Liver and heart weight are no longer attenuated, suggesting that these findings were due to starvation and not loss of CnAα in those tissues. Likewise, hypoglycemia is no longer evident in rescued mice which instead have mildly elevated glucose levels. In contrast, the gross kidney phenotype of null mice persists; glomeruli are abnormal, and kidney function is reduced. These data support our previous conclusion that CnAα is required for normal kidney development and function but also highlight the impact of early starvation on previous findings.

Rescued mice confirm much of our previous work showing that CnAα plays a significant role in kidney development and function. Small kidney size in the null mice appears to stem from a deficit of late nephrogenesis. While overall glomerular number is comparable and juxtamedullary nephrons appear typical, the majority of superficial cortical glomeruli are abnormal and likely non-functional. This is associated with tubular atrophy resulting in a decrease in cortical and OSOM volume and decreased kidney function. The mechanism by which CnAα affects nephrogenesis is still unclear.

Despite the success in rescuing the null mice at weaning, it is still possible that early nutritional deprivation complicates the phenotype of adult CnAα null kidneys. Although this is the first report of GFR in null mice, we did examine GFR in heterozygous mice previously [3] and found a mild decrease in GFR compared to WT littermates, supporting a primary causative role of CnAα rather than early nutritional deprivation. In addition, the similarities between CnAα−/− and COX-2−/− kidneys further supports a direct role for CnAα in nephrogenesis since COX-2 null mice are not reported to suffer from failure to thrive or reduced body weight [23], [24].

In addition to confirming altered kidney growth/development with loss of CnAα, new findings in this study include splenomegaly in CnAα null mice (**Table 2**). The increase in spleen size is likely due to an expansion of B cells and stromal cells as we reported previously in aged CnAα+/− mice [21]. It is unclear what cell type is responsible for changes in the spleen or if there are any significant phenotypic consequences as a result. These are interesting areas for future study.

The last new aspect of CnAα−/− phenotype that we found is mild hyperglycemia. Previously, we reported that null mice were hypoglycemic [3]. As a way to monitor re-feeding, we measured glucose levels in rescued mice and observed a rapid correction of hypoglycemia (likely the result of caloric restriction) with the modified diet. Both fasting and fed glucose levels are elevated in null mice; plasma insulin is slightly higher suggesting a normal compensatory response. Further testing revealed a mild defect in insulin sensitivity in null mice. Previous reports in the literature suggest that CnAα may have a direct role in insulin sensitivity in muscle [14], [25] and we were able to rule out gross abnormalities in the pancreas. A previous investigation into muscle changes with loss of CnAα or CnAβ reported a shift in the proportion of fiber types in skeletal muscle of CnAα−/− mice. In contrast to the kidney phenotype, which is maintained in rescued null mice, we find that muscle fiber types are normal. Instead, the decrease in insulin sensitivity may result from a reduction in IRS2 expression. Interestingly, there are limited reports that calcineurin inhibitors selectively target IRS2 and not IRS1 for enhanced degradation [26].

In conclusion, these studies help to differentiate between the effects of caloric deprivation and specific roles of CnAα in the kidney and also identify new aspects of CnAα action that warrant further investigation. Now that CnAα null mice can be rescued to adulthood – and are even fertile – new opportunities exist to verify previous data and to add to our knowledge of calcineurin action.
